# An improved high-throughput screening assay for tunicamycin sensitivity in *Arabidopsis* seedlings

**DOI:** 10.3389/fpls.2015.00663

**Published:** 2015-08-26

**Authors:** Maggie E. McCormack, Xiaoyu Liu, Melissa R. Jordan, Karolina M. Pajerowska-Mukhtar

**Affiliations:** Department of Biology, University of Alabama at Birmingham, Birmingham, ALUSA

**Keywords:** unfolded protein response, ER stress, high-throughput screening, tunicamycin, chlorophyll, *Arabidopsis thaliana*

## Abstract

Tunicamycin (Tm) sensitivity assays are a useful method for studies of endoplasmic reticulum stress and the unfolded protein response in eukaryotic cells. While Tm sensitivity and Tm recovery assays have been previously described, these existing methods are time-consuming, labor intensive, and subjected to mechanical wounding. This study shows an improved method of testing Tm sensitivity in *Arabidopsis* using liquid Murashige and Skoog medium versus the traditional solid agar plates. Liquid medium bypasses the physical manipulation of seedlings, thereby eliminating the risk of potential mechanical damage and additional unwanted stress to seedlings. Seedlings were subjected to comparative treatments with various concentrations of Tm on both solid and liquid media and allowed to recover. Determination of fresh weight, chlorophyll contents analysis and qRT-PCR results confirm the efficacy of using liquid medium to perform quantitative Tm stress assays.

## Introduction

Secretory proteins are synthesized and folded in the endoplasmic reticulum (ER) before being transported to target destinations. The protein folding capacity of the ER is balanced by its protein load and the amount of protein machinery available. When the ER stress is triggered by abiotic or biotic factors, the equilibrium shifts causing an overload of proteins unable to be posttranslationally processed with insufficient folding machinery. This accumulation of misfolded proteins activates the unfolded protein response (UPR) that initiates a variety of pathways to compensate and correct the ER equilibrium (Moreno and Orellana, 2011; Hollien, 2013). UPR is a highly conserved mechanism among eukaryotes and has direct roles in the systemic health of organisms. In mammals, UPR dysfunction can cause a variety of neurodegenerative, inflammatory, and immune disorders as well as cancer (Chakrabarti et al., 2011). In plants, UPR is induced by diverse environmental factors including pathogen infection and has been shown to play a role in the immune responses (Zhang et al., 2015). Likewise, yeast UPR is an essential pathway for responding to environmental stress such as heat shock and toxins (Sidrauski and Walter, 1997). UPR is part of a network of signaling cascades and involves the interruption of protein synthesis and translocation to the ER, the transcriptional upregulation of target genes including chaperones and folding enzymes, and, upon failure to reestablish equilibrium in the ER, the activation of apoptosis.

Inositol requiring enzyme 1 (IRE1)-mediated UPR pathway is evolutionarily conserved across eukaryotes, while additional UPR branches exist in mammals and plants. IRE1 is a type 1 transmembrane protein in the ER that regulates ER stress signal transduction and expression of UPR target genes (Iwata and Koizumi, 2012). Plants have two isoforms of IRE1, IRE1a, and IRE1b (Koizumi et al., 2001). Although IRE1a and IRE1b have overlapping functions in UPR (Chen and Brandizzi, 2012), both isoforms have distinct roles in responding to physiological and environmental conditions. IRE1a is preferentially involved in pathogen-induced UPR and has a higher induction under salicylic acid whereas IRE1b shows higher activation under *N*-glycosylation inhibitor tunicamycin (Tm), although both IRE1 isoforms share the same target mRNA and *ire1a-2 ire1b-4* double mutants exhibit a severely reduced ability to respond to ER stress (Moreno et al., 2012). In yeast, plants, and mammals, IRE1 directs unconventional splicing of its target transcripts encoding basic leucine zipper transcription factors, HAC1, bZIP60, and ATF4, respectively (Deng et al., 2013; Zhang et al., 2015). These transcription factors are processed upon ER stress, and the spliced mRNAs are translated and translocated to the nucleus to upregulate UPR target genes (Nagashima et al., 2011). IRE1 functions in association with ER-resident luminal binding protein (BiP) to transduce ER stress signal and express UPR target genes. There are three *BiP* genes in *Arabidopsis*, *BiP1*, *BiP2*, and *BiP3* (Noh et al., 2003). *BiP1* and *BiP2* encode homologous proteins that are ubiquitously expressed under normal conditions, whereas *BiP3* is specifically expressed under ER stress and is strongly induced by Tm (Maruyama et al., 2014). BiP2 and BiP3 have extensive and dynamic roles in mediating ER homeostasis in *Arabidopsis* (Liu et al., 2007a; Iwata et al., 2008; Zhang et al., 2015).

Tm, along with dithiothreitol (DTT), are chemical inducers of UPR commonly used in the laboratory. Tm is shown to trigger ER-mediated stress in diverse eukaryotic organisms including plants, human, and yeast (Hauptmann et al., 2006; Iwata et al., 2010; Oslowski and Urano, 2011). Tm prevents *N*-linked glycosylation (formation of *N*-glycans) by interrupting the enzyme GlcNac phosphotransferase responsible for the initial steps in glycosylation (Koizumi et al., 1999). *N*-glycans are essential for proper protein folding and stability as well as transport to the Golgi apparatus for vesicle packaging and secretion. In mammals, *N*-glycans have important functions in immune cells, including trafficking, cell signaling, and effector binding (Maverakis et al., 2015). In plants, *N*-glycans are also implicated in the postrranslational modification of microbe-associated molecular patterns (MAMPs) receptors and immune response (Saijo et al., 2009; Häweker et al., 2010). DTT is a strong reducing agent that induces acute ER stress. Although a potent ER stress inducer, DTT is not an ideal UPR-triggering agent for *in vivo* studies since it inhibits disulfide bond formation during protein formation in the ER and cytosol alike, making it non-specific to ER stress. Additionally, Tm and DTT differentially affect the kinetics of ER stress and may influence UPR target gene expression (Li et al., 2011).

High-throughput methods for studies of UPR have previously been developed for mammals and yeast (Back et al., 2005). These methods employ the use of pharmacological chemicals such as Tm and DTT, and others, as well as reporter genes and genetic screening. Yeast is a relatively simple organism to activate UPR, as chemicals can be directly added to solid or liquid plates and influence growth of colonies. Recently, there has been a need for more sensitive assays to elucidate the dynamic and progressive homeostatic roles of UPR. In yeast, the use of fluorescent markers attenuates the changes in UPR during nonlethal ER stress across a range of chemical doses (Pincus et al., 2010). Coupled with high-throughput techniques such as flow cytometry and cell sorting, these methods allow for the observation of subtle changes in cellular environments and transcriptional changes during various temporal phases of UPR. Human pancreatic cell lines and mouse embryonic fibroblasts are common cell cultures used in studying ER stress and UPR and have been used in conjunction with high-throughput methods such as flow cytometry (McCullough et al., 2001; Pino et al., 2009). However, in plants these methods are less feasible because protoplast preparation is laborious and has the potential for providing false data as the cells lack cell walls and are often stressed (Jiang et al., 2013). Although protoplast isolation methods have experienced several improvements over the last decade (Yoo et al., 2007; Wu et al., 2009), the protocols for these techniques are still limited by the fragility of the protoplast cultures and their ability to assay sensitive genotypes during cellular stress across several timepoints. Thus, there is a need for high-throughput methods for assaying stress responses using intact seedlings until cell culture procedures can be further optimized.

Tm sensitivity assays have been used to elucidate UPR mechanisms and key players as well as UPR’s role in immune responses and cell death in plants (Iwata et al., 2010; Jaspers and Kangasjärvi, 2010; Ozgur et al., 2014). Traditionally, Tm sensitivity assays are performed on seedlings grown on solid agar Murashige and Skoog (MS) plates and involve either scoring direct germination on plates supplemented with Tm or observing the recovery of seedlings exposed to various concentrations Tm for a period of time and subsequently returned to normal conditions. Germinating seeds in the presence of Tm, however, affects embryo development because the embryos do not have the cellular infrastructure in place to cope with extensive chemical stress. Tm can lead to embryo arrest resulting in low germination rates for some sensitive genotypes (Cordewener et al., 1991; Mishiba et al., 2013), thus it is not an ideal measure of the UPR or physiological response to ER stress inducers. The germination assay is especially problematic for hypersensitive genotypes that may fail to germinate. Although the recovery assay presents more favorable conditions for observing ER stress response, the use of solid media has several limitations. The physical transfer of seedlings between MS plates and Tm plates has the potential for wounding that may introduce additional stress to seedlings, which in turn may affect the pathophysiological response to Tm. The exposure of the seedlings to the Tm supplemented media is non-uniform on solid media and may not induce a reliable and reproducible stress response. Together these factors introduce variables that make quantifying data from Tm stress assays on solid media ambiguous and unreliable.

To circumvent these caveats of the solid media assay and adapt it to large-scale screening, we designed an advanced method for quantitative assessment of UPR sensitivity or tolerance in *Arabidopsis*. Here we report an improved Tm sensitivity assay with the use of liquid MS medium intended to minimize damage and unwanted stress to seedlings. The use of liquid medium rather than solid agar plates allows for the manipulation of the medium itself over the seedlings, which additionally saves materials and space. We also demonstrate that the assay is adaptable to screening of *Arabidopsis* mutants and offers a quantitative output in the form of fresh weight of seedlings after Tm exposure and chlorophyll content of the leaves as indicators of overall plant health. Our experimental framework can be expanded to high-throughput screens involving large numbers of genotypes.

## Materials and Methods

### Plant Lines used in this Study

Wild type Col-0 plants were obtained from the *Arabidopsis* Biological Resource Center. *ire1a-2 ire1b-4* double mutant plants were reported previously (Moreno et al., 2012). *ire1a-2* (SALK_018112) is in Col-0 background and *ire1b-4* (SAIL_238_F07) is in Col-3 background. *bip2-1 bip3-1* double mutant was kindly provided by Dr. Shuh-ichi Nishikawa (Maruyama et al., 2010). *bip2-1* (SAIL_1153_D09) is in Col-3 background and *bip3-1* (SALK_024133) is in Col-0 background.

### Tm Stress Assays (Solid/Liquid)

**FIGURE 1:**
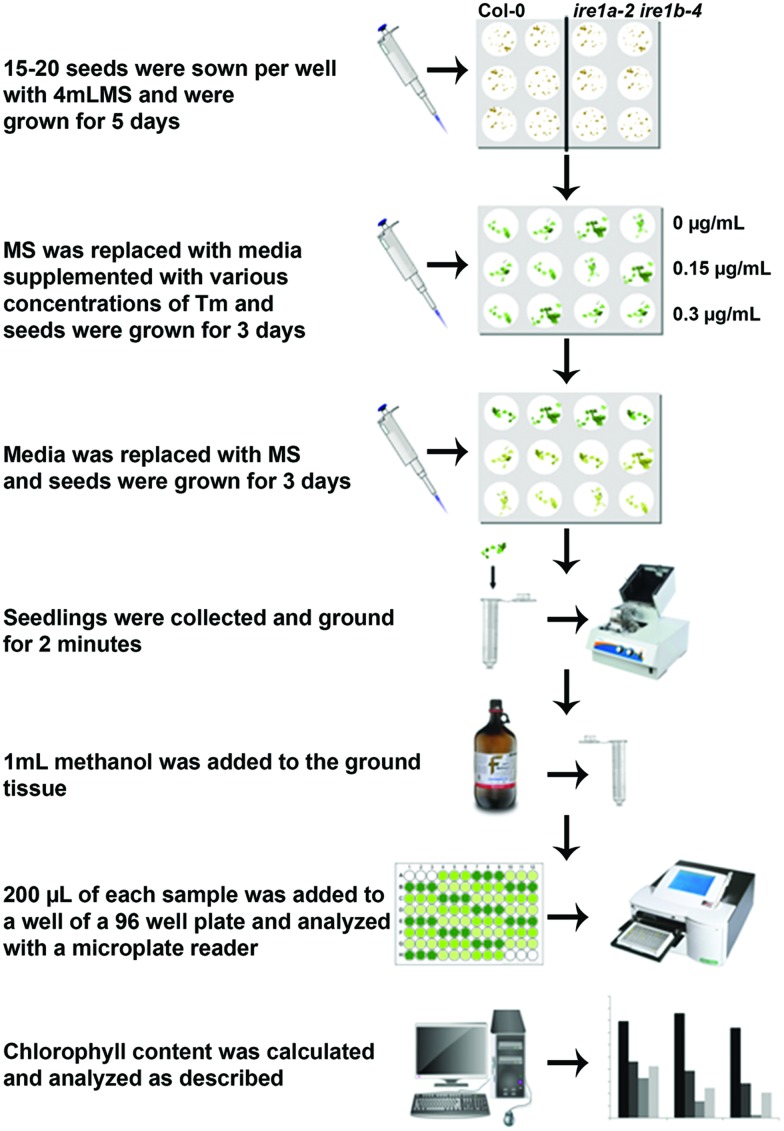
**Flowchart of the procedures to quantify tunicamycin (Tm) sensitivity of *Arabidopsis* seedlings using liquid media followed by chlorophyll measurement**.

Seeds were washed with a solution of 0.05% Triton and 70% Ethanol then stratified for 4 days at 4°C. For the assay on solid plates, seeds were placed in sterile 0.1% agar solution and pipetted onto solid Murashige Skoog (MS) growth medium (Phytotechnology Lab, Overland Park, KS, USA) with 1.2% sucrose (Fisher) and 50 mg/L ampicillin (Fisher) and allowed to grow for 5 days at 22°C under a 16 h light/8 h dark (for the initial dose-response assay) or 12 h light/12 h dark (remaining experiments) photoperiod. 15 seeds were transferred to solid MS medium containing either 0.15 or 0.3 μg/mL Tm, (Sigma-Aldrich T7765) and allowed to grow for 3 days at 22°C. The treated seeds were then transferred back to solid MS medium plates and allowed to grow for 3 days before collection for chlorophyll analysis. For the assay in liquid media, seeds were placed in sterile 0.1% agar solution and 15–20 seeds were pipetted into each well of a 12-well polystyrene plate (Fisher) containing 4 mL 0.5 × MS medium with 0.6% sucrose and ampicillin and grown for 5 days at 22°C. The MS medium was removed and replaced with fresh MS media or media containing either 0.15 or 0.3 μg/mL Tm and allowed to grow for 3 days at 22°C. The medium was again replaced with fresh MS medium and plants were allowed to grow for 3 days before collection for chlorophyll analysis. The experimental design is outlined in **Figure 1**.

### Fresh Weight Determination

Ten seedlings per genotype were collected 3 days after recovery from treatment with 0 μg/mL (MS only), 0.15 μg/mL, or 0.3 μg/mL Tm. Seedlings were weighed immediately following harvest and weights were recorded in mg. Data were subjected to statistical analyses (Tukey’s HSD test) using SAS 9.3 software package (SAS Institute, Cary, NC, USA).

### Chlorophyll Extraction and Analysis

Five leaf samples were collected from *Arabidopsis* 3 days after recovery from treatment with 0 μg/mL (MS only), 0.15 μg/mL, or 0.3 μg/mL Tm. The leaves were frozen in liquid nitrogen and homogenized in a high-throughput homogenizer (Powergen High Throughput Homogenizer, Fisher), then washed with 1 mL methanol, homogenized for 2 min, and centrifuged for 2 min at 13,000 g. Chlorophyll was measured by adding 200 μL of supernatant to a 96-well polystyrene plate (Fisher) and read using a microplate reader (Epoch, Biotek) at 652 and 665 nm wavelengths. Absorbance corrections and chlorophyll calculations were completed as described in Ritchie (2006). Data were subjected to statistical analyses (Tukey’s HSD test) as described above.

### RNA Extraction and qRT-PCR

Five *Arabidopsis* seedlings were collected 3 days after Tm treatment (Tm stress) and after additional 3 days of recovery in plain MS (Tm recovery). Plant tissues were frozen in liquid nitrogen and homogenized through the high-throughput homogenizer. Total RNA was extracted using the TRIzol reagent (Invitrogen) and RNA concentration was measured with the BioPhotometer Plus (Eppendorf). To remove the genomic DNA contamination, DNase I (Ambion) treatment was performed for each sample. The cDNA was generated by reverse transcription through the SuperScript III first-strand RT-PCR kit (Invitrogen). The relative transcript abundance of the UPR-responsive genes was determined using GoTaq qPCR Master Mix (Promega) in a RealPlex S MasterCycler (Eppendorf). Gene specific primers used in this study are: *CRT1*, *CRT3*, *CNX1*, *bZIP60* splicing activity, and *UBQ5* as a control. The following primers were used in this study: CRT1_F 5′CTGTGGTGGTGGCTAC 3′; CRT1_R 5′ GTCTCACATGGGACCT 3′; CRT3_F 5′ ATGACCCCAACGATGT 3′; CRT3_R 5′ CCTTGTAGTTCGGGTTCT 3′; CNX1_F 5′ CCCATGTCTCCGC 3′; CNX1_R 5′ CACGGCATTTGGATCAG 3′; UBQ5_F 5′ GTAAACGTAGGTGAGTCC 3′; UBQ5_R 5′ GACGCTTCATCTCGTCC 3′. The bZIP60u (unspliced) and bZIP60s (spliced) transcripts were quantified using sequence-specific primers bZIP60_F 5′GGAGACGATGATGCTGTGGCT 3′; bZIP60u_R 5′ CAGGGATTCCAACAAGAGCACAG 3′; bZIP60s_R 5′ CAGGGAACCCAACAGCAGACT 3′ through quantitative real-time RT-PCR as described previously (Moreno et al., 2012), and the induction fold of Tm over mock treatment (H_2_O) was calculated. The bZIP60 splicing activity at each time point was determined by normalizing induction fold of bZIP60s against bZIP60u. Data were subjected to statistical analyses (Student’s *t*-test) using SAS 9.3 software package (SAS Institute, Cary, NC, USA).

## Results

**FIGURE 2:**
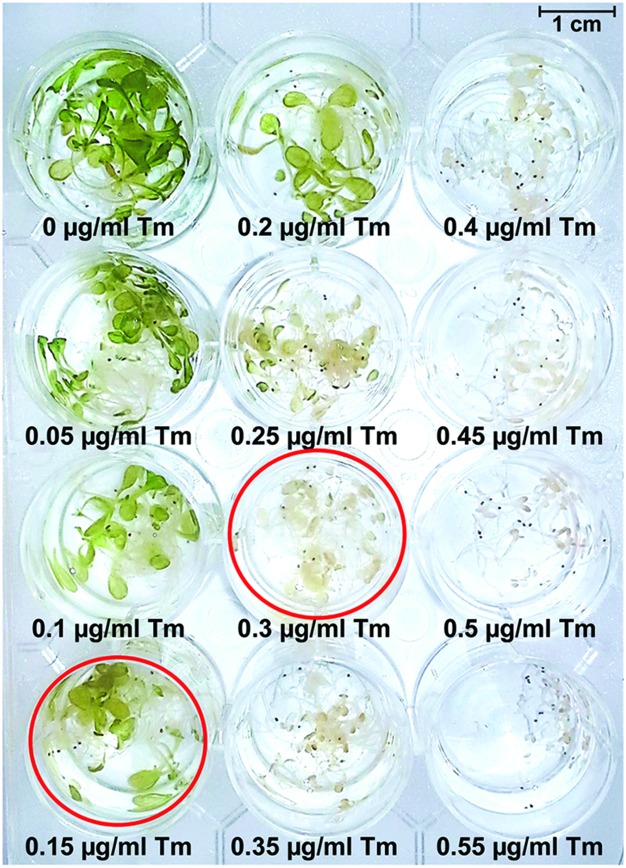
**Dose-response assay to determine Tm concentrations resulting in appearance of retarded growth and chlorosis in *Arabidopsis* seedlings.** Long-day grown 5-day-old seedlings were subjected to Tm treatment for 3 days and photographed. Wells corresponding to Tm concentrations of 0.15 and 0.3 μg/mL are circled in red.

It is well established that Tm treatment can be conducted on solid MS media and leads to a stress response that is equivalent to UPR (Iwata and Koizumi, 2005; Oslowski and Urano, 2011). The most frequently used concentrations of Tm on solid plates range between 0.1 and 0.5 μg/mL (Iwata and Koizumi, 2005; Pajerowska-Mukhtar et al., 2012). However, liquid MS culture of *Arabidopsis* seedlings offers a more uniform delivery of the chemical compound to the plant and is a widespread method to test responses to chemicals (Schreiber et al., 2008). We set out to adapt the liquid assay to quantify Tm sensitivity through a recovery assay. First, we subjected 5-day-old wild type Col-0 seedlings to increasing doses of Tm in liquid culture (**Figure 2**) for 3 days followed by replacing the MS medium containing Tm with fresh aliquot of regular MS medium. We observed progressively smaller and more chlorotic seedlings as the Tm concentrations increased, with a nearly complete arrest in growth and development and profound bleaching at concentrations of 0.4 μg/ml and above. Based on this dose-response experiment, we determined that under liquid MS conditions the Tm concentrations of 0.15 and 0.3 μg/ml can serve as appropriate conditions to test for severe and mild defects in the UPR tolerance, respectively.

**FIGURE 3:**
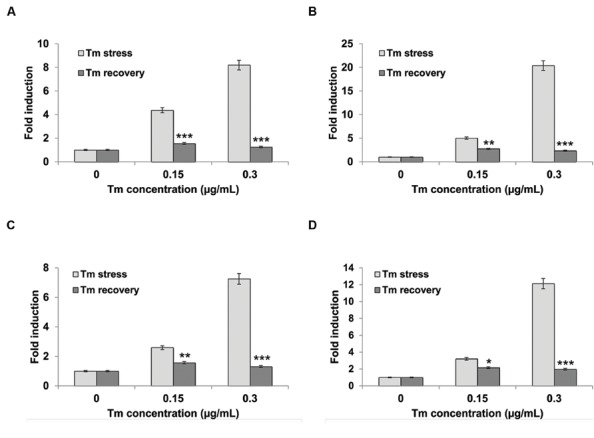
**Unfolded protein response (UPR) target genes are upregulated during Tm-induced ER stress.** Five-day-old *Arabidopsis* seedlings were pre-grown in liquid MS medium, then subjected to stress by replacing the media with liquid MS supplemented with designated concentrations of Tm for 3 days. The recovery was performed by incubating stressed seedlings in liquid MS medium for another 3 days. The transcript accumulation of **(A)** *CRT1*, **(B)** *CRT3*, **(C)** *CNX1*, and **(D)** *bZIP60* splicing were measured by real-time PCR and normalized to a housekeeping gene *UBQ5*. Statistical analysis was performed with Student’s *t*-test comparing values from Tm-exposed seedlings to regular MS-grown seedlings, *p < 0.05, **p < 0.01, ***p < 0.001. Experiments were performed in at least three independent biological replications. Error bars represent SE between these replications.

To firmly establish that the phenotypic responses of seedlings grown on Tm are indeed caused by the UPR stress, we next tested expression levels of several canonical ER stress marker genes. We treated liquid MS-grown 5-day-old wild type Col-0 seedlings with 0.15 μg/mL and 0.3 μg/mL Tm for 3 days, then replaced the media with plain MS and continued to culture the seedlings for another 3 days. We sampled the seedlings at 3-days post Tm treatment (Tm stress) and after the 3-days recovery period (Tm recovery). We used qRT-PCR (quantitative Reverse Transcription PCR) to determine levels of transcriptional induction of several UPR stress markers: *Calreticulin 1* (*CRT1*), *CRT3*, and *Calnexin 1* (*CNX1* **Figures 3A–C**). Calreticulins (CRTs) are ER-localized proteins that bind Ca^2++^ and function in the proper folding of proteins through oligosaccharide modification. *Arabidopsis* contains three isoforms of CRT: CRT1, CRT2, and CRT3. All three family members are essential for the ER stress response (Wang et al., 2005; Christensen et al., 2008; Saijo et al., 2009; Pajerowska-Mukhtar et al., 2012) and function in many other signaling pathways, including defense against pathogen infection (Christensen et al., 2010). Calnexin (CNX) is also an ER-localized chaperone that monitors protein folding activity within the ER. Specifically, the function of CNX is to retain misfolded *N*-linked glycoproteins. *Arabidopsis* CNX was demonstrated to be implicated in pathogen defense (Wang et al., 2005). The seedlings exhibited a marked increase in UPR marker genes at 3-days post exposure to both concentrations of Tm (**Figure 3**). The amplitude of induction ranged from 2.5 to 5-fold for 0.15 μg/ml Tm and 7–20-fold for the higher 0.3 μg/ml Tm dose. Thus, we confirmed that the liquid assay can reliably induce UPR marker genes in *Arabidopsis* seedlings. Next, we asked whether the recovery in plain MS media will translate into a deactivation of the UPR response. Following a 3-days period of incubation under stress-free conditions, the ER stress markers expression decreased dramatically compared to samples collected directly after Tm exposure, and reverted to nearly basal levels. This observation confirms that the wild type Col-0 seedlings can effectively recover from the ER stress under the conditions tested (**Figures 3A–C**). To further corroborate these data, we also measured the activity of *bZIP60* splicing, which reflects activation of the IRE1 signaling branch of UPR, using our previously established qRT-PCR assay (Moreno et al., 2012). We observed a 2.5 and 12-fold increase in *bZIP60* splicing in the samples treated with Tm doses of 0.15 and 0.30 μg/ml, respectively. This *bZIP60* splicing activity is specific to Tm-induced ER stress as we did not observe an induction of *bZIP60* splicing in the Tm recovery samples (**Figure 3D**). Taken together, we conclude that Tm can specifically induce UPR stress in *Arabidopsis* grown in liquid MS medium.

To extend our system to testing *Arabidopsis* genotypes other than Col-0, we selected one single mutant and two double mutants that have defects in ER stress perception and/or signaling. We employed *ire1b-4* single mutant and *ire1a-2 ire1b-4* and *bip2-1 bip3-1* double mutant plants, all of which have been previously demonstrated to be sensitized to Tm application while grown on solid MS media and thus constitute important controls for our assay (Chen and Brandizzi, 2012; Mishiba et al., 2013). The *ire1a-2 ire1b-4* double mutant exhibits an extreme phenotype under Tm stress compared to their respective *ire1a* and *ire1b* single mutants, along with an increase in cell death (Mishiba et al., 2013). BiPs are essential ER proteins that function in protein translocation and folding and are upregulated during UPR. BiPs are also involved in the activation of both arms of plant UPR, namely the transmembrane proteins IRE1a and IRE1b branch as well as bZIP17- and bZIP28-mediated pathway (Liu et al., 2007b; Che et al., 2010; Liu and Howell, 2010; Srivastava et al., 2013).

To demonstrate the advantage of our newly improved liquid culture assay, we sowed 15–20 seeds of Col-0, *ire1b-4*, *ire1a-2 ire1b-4*, and *bip2-1 bip3-1* side by side in 12-well plates containing liquid MS medium and on solid MS plates. We allowed the seeds to germinate and continue their growth for 5 days. At this point, the seedlings grown on solid MS plates were manually transferred using a pair of tweezers onto freshly poured plates containing MS media supplemented with 0.15 or 0.3 μg/mL Tm. For the liquid MS-grown seedlings, media was discarded using a regular pipette and replaced with fresh media supplemented with 0.15 or 0.3 μg/mL Tm. No physical stress to the seedlings occurred at any step of the liquid assay procedure. After 3 days of Tm exposure, plate-grown seedlings were once again handled mechanically to transfer onto regular MS plates, while for the liquid-grown plates media was once again replaced with fresh liquid MS. For both conditions, seedlings were allowed 3 days recovery before collection for downstream analyses (**Figure 1**).

**FIGURE 4:**
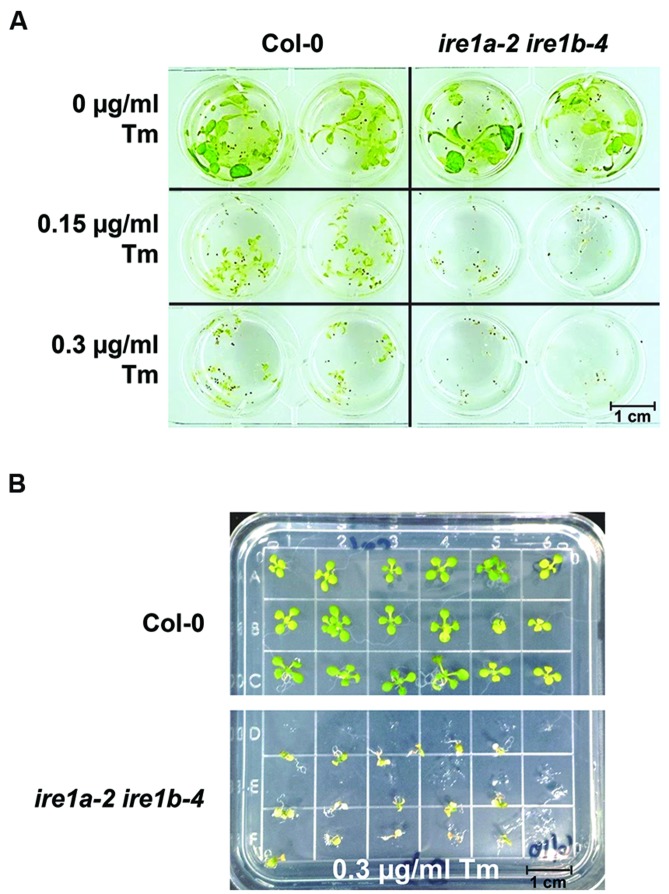
**Phenotypic responses of *Arabidopsis* wild type Col-0 and *ire1a-2 ire1b-4* seedlings to varied concentrations of Tm. (A)** Seeds were grown in liquid MS media for 5 days, then the media was removed and replaced by fresh MS supplemented with designated concentrations of Tm for 3 days. Phenotypic responses, such as dwarfism and chlorosis, were documented. The *ire1a-2 ire1b-4* plants display more profound stress symptoms compared to Col-0 under both Tm concentrations. **(B)** Seeds were grown on solid plates under the same conditions as seeds grown in **(A)**. Seeds were transferred to plates containing various concentrations of Tm after 5 days of growth on MS and were transferred again to MS plates after 3 days of growth on plates supplemented with Tm. Seeds were allowed 3 days recovery before data analysis.

**FIGURE 5:**
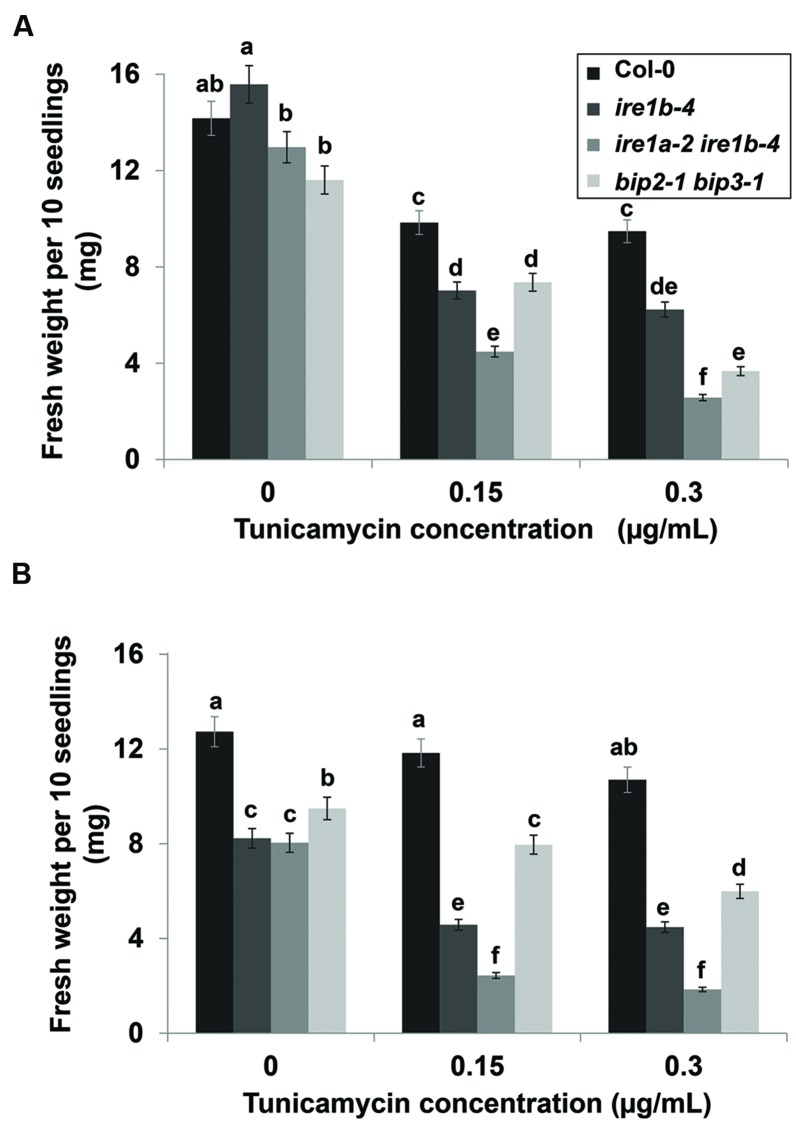
**Fresh weight of seedlings after Tm treatment. 10 seedlings per genotype were weighed after 3 day recovery from various concentrations of Tm in liquid **(A)** and solid **(B)** MS media.** Error bars represent SE of the means. Statistical analysis was performed with Tukey’s HSD test. Bars within a class connected by the same letter did not differ from each other at *p* < 0.05. Experiments were performed in at least three independent biological replications.

Two primary phenotypes of Tm-stressed seedlings are a decrease in their size and visible yellowing and chlorosis (**Figures 2** and **4A,B**). A combined quantitative assessment of these two plant responses offers detailed insights in the level of sensitivity to UPR stress. After 3 days of recovery from Tm exposure, 10 seedlings per genotype were collected and weighed from both liquid and solid plates (**Figure 5**). We observed progressively decreased weight of liquid-grown Col-0 following recovery after exposure to 0.15 and 0.3 μg/mL concentrations of Tm, indicating an induction of Tm-dependent UPR (**Figure 5A**). However, Col-0 seedlings weighed 2–5 times more than *ire1a-2 ire1b-4* double mutant seedlings at 0.15 and 0.3 μg/mL concentrations of Tm, respectively. This indicates that the *ire1a-2 ire1b-4* double mutant exhibits a decreased ability to cope with and recover from UPR, consistent with the previous findings (Chen and Brandizzi, 2012; Moreno et al., 2012). As expected, *ire1a-2 ire1b-4* double mutants weighed nearly two times less than *ire1b-4* mutants. Similarly, *bip2-1 bip3-1* double mutants weighed two times less than *ire1b-4* and 3 times less than Col-0 at the higher dose of Tm (**Figure 5A**). However, the reduction in the fresh weight observed on solid MS plates under the conditions tested, although statistically significant, was not as dramatic compared to liquid assay. This was true for all the genotypes included in our assay and in particular the *bip2-1 bip3-1* double mutants (**Figure 5B**). This effect can be, at least in part, attributed to the non-uniform Tm exposure on solid plates, which is a known limitation of this assay, especially since these mutants have previously been shown to have severe phenotypic responses to Tm (Noh et al., 2003). In addition, it is worth noting that all genotypes grown on solid plated were smaller than their counterparts cultured in liquid MS medium (**Figures 5A,B**).

**FIGURE 6:**
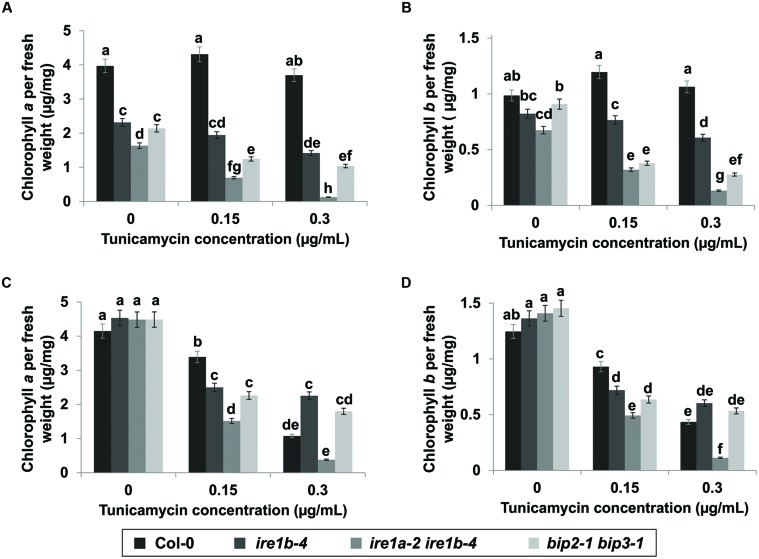
**Chlorophyll *a* and *b* content after Tm treatment.** Chlorophyll a was extracted with methanol from seedlings 3 days after exposure to various concentrations of Tm in liquid **(A)** and solid **(B)** media. Chlorophyll *b* was extracted following the same procedure in liquid **(C)** and solid **(D)** media. Chlorophyll *a* and *b* content was read using a microplate reader at 652 and 665 nm wavelengths. Error bars represent standard error of the means. Statistical analysis was performed with Tukey’s HSD test. Bars within a class connected by the same letter did not differ from each other at *p* < 0.05. Experiments were performed in at least three independent biological replications.

To substantiate the fresh weight data, we next quantified seedling chlorosis through a quantitative measurement of chlorophylls *a* and *b* contents per unit of fresh weight resulting from Tm exposure on both liquid MS medium and on solid MS plates. Seedlings were collected after 3 days of recovery from Tm treatment and analyzed for chlorophyll accumulation at 652 and 665 nm wavelengths using a microplate reader. Col-0 showed 30 times higher chlorophyll *a* and *b* contents than the *ire1a-2 ire1b-4* double mutant in liquid culture (**Figures 6A,C**). The lowered chlorophyll contents per unit of fresh weight in Col-0 seedlings treated with 0.3 μg/mL Tm on solid medium might be attributed to chlorosis without a substantial decrease in fresh weight (**Figures 5B and 6B,D**). It is also plausible that this decrease in chlorophyll contents was caused by mechanical damage to the fragile seedlings during transfer between plates. The *ire1a-2 ire1b-4* showed severe reduction of chlorophyll *a* and *b* content on both liquid and solid plates. Chlorophyll *a* content was 11–13 times lower in *ire1a-2 ire1b-4* mutants compared to *ire1a-2 ire1b-4* controls and 5–11 times lower compared to *ire1b-4* single mutants treated with 0.3 μg/mL Tm, indicating the sensitivity of the *ire1a-2 ire1b-4* double knock-out as well as the severity of chlorosis at higher Tm doses. The reduction of chlorophyll *b* content showed a similar although less severe pattern, which is consistent with chlorophyll *a* being the dominant chlorophyll pigment accumulated by leaf tissue. The *bip2-1 bip3-1* double mutants showed a similar trend in chlorophyll reduction comparable to that of *ire1a-2 ire1b-4*, although less dramatic (**Figures 6A–D**). The redundancy of BiP1 and BiP2 may contribute to the slightly higher levels of chlorophyll in the *bip2-1 bip3-1* plants compared to *ire1a-2 ire1b-4* double mutants. Altogether, these results indicate the effectiveness of using liquid medium to induce UPR through Tm treatment and highlight the numerous advantages of using this assay for large scale screens.

## Discussion

The ER plays a role in many secretory pathways in eukaryotic cells. The capacity of the ER to adjust to fluctuating environmental conditions is essential for the cell’s ability to respond to various internal and external stresses, such as heat/cold shock, pathogen infection, and drought. The UPR attempts to restore ER homeostasis by reducing protein translocation to the ER and upregulating genes involved in the synthesis of chaperones and protein folding machinery. Although UPR’s role in the pro-death cellular response to stress is little understood, it has significant implications in immune responses and various disease states.

The improved Tm assay described here may facilitate quantitative analysis of ER stress and UPR in various physiological applications through its ability to screen multiple genotypes while reducing stress from mechanical damage and transplanting. Liquid medium poses less mechanical damage to seedlings by averting the need to handle the seedlings directly. This feature is especially beneficial when testing extremely sensitive genotypes such as the *ire1a-2 ire1b-4* double mutant. Liquid medium may offer more robust results in this respect, as the stress responses are less likely to be caused by unintended factors. Coupled with high-throughput methods such as chlorophyll analysis and qRT-PCR, assaying for Tm sensitivity may facilitate our understanding of UPR’s role in maintaining plant health through a multitude of pathways. The improvements described in this study provide increased efficiency that meets the growing demand for high-throughput techniques.

Eliminating agar has multifold significance in ensuring more reliable data, as it allows bypassing several disadvantageous effects of solid medium. First, the composition of agar batches varies and can affect experimental outcomes by influencing growth patterns (Jain et al., 2009). This non-uniformity can also affect exposure to chemical treatments such as Tm and subsequently fail to induce normally activated downstream effects. These issues are especially problematic with *Arabidopsis* seedlings as the seeds grow out of the medium and restrict the available surface area and tissue types that are in direct physical contact with the medium and supplemented chemicals. When using seedlings grown on solid medium for quantitative analysis, the medium itself may interfere with measurements by adhering to tissue, which affects the weight of the seedlings, or by causing damage to the seedlings when the lateral roots become embedded in the agar and cause breakage when seedlings are removed.

Mechanical damage can affect experimental results by causing additional stress. This is especially problematic for hypersensitive genotypes that may already be extremely stressed while entering into the experimental assay. Wounding can induce additional gene expression changes (Reymond et al., 2000; León et al., 2001; Walley et al., 2007) that constitute a broad defense response. Mechanical stress has also been shown to result in an accumulation of reactive oxygen species (ROS) in leaf tissue (Orozco-Cardenas and Ryan, 1999) as well as the systemic activation of jasmonic acid (JA) synthesis that is analogous to the inflammatory response in mammals (Bergey et al., 1996; Howe and Schilmiller, 2002; Li et al., 2005; Schilmiller and Howe, 2005). ROS and JA signaling are sustained for several hours after the initial wounding and alter defense and metabolic pathways. ROS is an important component of several defense pathways (Torres et al., 2006) and is involved in the hypersensitive response (Van Breusegem and Dat, 2006). Cells mediate ROS levels and oxidative damage through several mechanisms (Alscher et al., 2002; Vanderauwera et al., 2011), including the degradation of chloroplasts (Kariola et al., 2005). JA is the primary hormonal response to wounding and is involved in an intricate network of crosstalk with other defense hormones ethylene and salicylic acid (Reinbothe et al., 2009). Among the genes induced by JA are proteinase inhibitors that target a wide range of protein classes (Pearce et al., 1991; Mosolov and Valueva, 2005; Hartl et al., 2011). The potential for mechanical trauma during stress assays has led many researchers to move toward simplified methods of handling seedlings that reduce unwanted downstream effects of wounding as well as offer improved large-scale quantitative analysis. A common feature of many of these novel protocols involves the shift toward liquid medium (Conn et al., 2013; Alatorre-Cobos et al., 2014) as it is less labor intensive, more time efficient, easier to quantify, and substantially more cost-effective than solid media assays. Liquid medium has been shown to deliver excellent results for several species of model plants (Battke et al., 2003; Conn et al., 2013) and a variety of chemical treatments (Lichter, 1981; Schreiber et al., 2008). The assay described here illustrates a continuation in improving standards for sensitivity assays using *Arabidopsis* seedlings.

## Author Contributions

MM carried out the stress assays and chlorophyll analysis and drafted the manuscript. XL performed RNA extraction and qRT-PCR analysis. MJ optimized the experimental methods. KP-M designed the study, coordinated its completion, and drafted the manuscript. All authors read and approved the final manuscript.

## Conflict of Interest Statement

The authors declare that the research was conducted in the absence of any commercial or financial relationships that could be construed as a potential conflict of interest.
